# Non-coding RNAs: emerging biomarkers and therapeutic targets in cancer and inflammatory diseases

**DOI:** 10.3389/fonc.2025.1534862

**Published:** 2025-03-10

**Authors:** Basma Hossam Abdelmonem, Lereen T. Kamal, Lilian Waheed Wardy, Manon Ragheb, Mireille M. Hanna, Mohamed Elsharkawy, Anwar Abdelnaser

**Affiliations:** ^1^ Institute of Global Health and Human Ecology, School of Sciences and Engineering, The American University in Cairo, New Cairo, Egypt; ^2^ Basic Sciences Department, Faculty of Physical Therapy, October University for Modern Sciences and Arts (MSA), Giza, Egypt; ^3^ Biotechnology Graduate Program, School of Sciences and Engineering, The American University in Cairo, New Cairo, Egypt; ^4^ Research and Development Department, Eva Pharma for Pharmaceuticals Industries, Cairo, Egypt; ^5^ School of Medicine, New Giza University (NGU), Giza, Egypt

**Keywords:** ncRNA, miRNA, lncRNA, circRNA, cancer, inflammation, biomarkers, therapy

## Abstract

Non-coding RNAs (ncRNAs) have a significant role in gene regulation, especially in cancer and inflammatory diseases. ncRNAs, such as microRNA, long non-coding RNAs, and circular RNAs, alter the transcriptional, post-transcriptional, and epigenetic gene expression levels. These molecules act as biomarkers and possible therapeutic targets because aberrant ncRNA expression has been directly connected to tumor progression, metastasis, and response to therapy in cancer research. ncRNAs’ interactions with multiple cellular pathways, including MAPK, Wnt, and PI3K/AKT/mTOR, impact cellular processes like proliferation, apoptosis, and immune responses. The potential of RNA-based therapeutics, such as anti-microRNA and microRNA mimics, to restore normal gene expression is being actively studied. Additionally, the tissue-specific expression patterns of ncRNAs offer unique opportunities for targeted therapy. Specificity, stability, and immune responses are obstacles to the therapeutic use of ncRNAs; however, novel strategies, such as modified oligonucleotides and targeted delivery systems, are being developed. ncRNA profiling may result in more individualized and successful treatments as precision medicine advances, improving patient outcomes and creating early diagnosis and monitoring opportunities. The current review aims to investigate the roles of ncRNAs as potential biomarkers and therapeutic targets in cancer and inflammatory diseases, focusing on their mechanisms in gene regulation and their implications for non-invasive diagnostics and targeted therapies. A comprehensive literature review was conducted using PubMed and Google Scholar, focusing on research published between 2014 and 2025. Studies were selected based on rigorous inclusion criteria, including peer-reviewed status and relevance to ncRNA roles in cancer and inflammatory diseases. Non-English, non-peer-reviewed, and inconclusive studies were excluded. This approach ensures that the findings presented are based on high-quality and relevant sources.

## 1 Introduction

In recent years, there has been a significant expansion in the era of RNA biology, showing the sophisticated mechanisms of gene regulation that extend far beyond the conventional protein-coding genes. While messenger RNAs (mRNAs) are transcribed and translated into proteins, most of the genome is transcribed into RNA molecules known as non-coding RNAs (ncRNAs) that do not ultimately translate into proteins (1). The major classes of ncRNAs that have gained increased research attention are microRNAs (miRNAs), long non-coding RNAs (lncRNAs), and circular RNAs (circRNAs) (2). Each class has unique structural and functional properties. ncRNAs have an essential role in the regulation process of gene expression in various biological pathways through the interaction with DNA, other RNAs, and proteins to modify gene activity. The regulatory functions of ncRNAs are portrayed at different levels, either transcriptional, post-transcriptional, or even epigenetic regulation (3). Their regulatory roles can be transcriptional in correlation to chromatin-modifying complexes to activate or silence target genes. The regulation can otherwise be post-transcriptionally through binding to complementary regions to control protein production. ncRNAs can also be involved in epigenetics, including DNA methylation and histone modification, which alter the expression of certain genes (3). Other ncRNAs influence the splicing procedure, ultimately modulating protein expression. Thus, ncRNAs are found in complex regulatory networks, interacting with several players to control gene expression, specifically in cancer and inflammatory disorders (4).

The process of carcinogenesis involves several steps in which genetic and epigenetic changes occur in normal cells (5). Epigenetic changes have been gaining much more attention because ncRNAs have been found to act as oncogenes or tumor suppressors to form complex networks of mutual interactions and modulate several signaling pathways (6). As a result, ncRNAs become promising targets for diagnosis, prognosis, and treatment. Understanding how cancer develops has been greatly enhanced by the discovery of ncRNAs, which provide information about the role of the entire genome in this process (7). A major obstacle in contemporary biology remains understanding their function in carcinogenesis, as the roles and mechanisms of the majority of ncRNAs are still unclear (8, 9).

There is a strong link between cancer and inflammation in which chronic inflammation is a stimulator of cancer development. Cancer cells interact with surrounding pro-inflammatory and immune cells, forming the inflammatory tumor microenvironment (TME) (10, 11). Chronic inflammation releases pro-inflammatory and oncogenic mediators like nitric oxide, cytokines (IL-1β, IL-2, IL-6, TNF-α), growth factors, and chemokines, creating a TME conducive to tumorigenesis (12). These key mediators facilitate intercellular communication in the TME, linking chronic inflammation to cancer by activating oncogenic pathways and enhancing immune evasion where cancer cells exploit the tolerogenic functions of monocytes, T regulatory cells (Tregs), and B regulatory cells (Bregs), resulting in local or systemic immunosuppression (13, 14). For this reason, inflammation is a key feature of cancer, with elevated inflammatory mediators linked to poor patient prognosis (15). Inflammation from prolonged infections is linked to oncogenesis, with studies showing that many cancer-related deaths are tied to chronic unresolved infections (16). Ongoing studies explore anti-inflammatory and antitumor therapies leveraging these immune responses. Among the emerging areas of interest, ncRNAs have gained considerable attention due to their remarkable potential to regulate key molecular pathways involved in these diseases (17, 18).

There are several FDA-approved ncRNA therapeutics, with many more in clinical trials specific to cancer and inflammatory diseases (19). Still, several are in various stages of clinical trials with promising results concerning their safety and efficacy. There are a variety of different strategies applied to overcome challenges that are faced by those therapeutics, like the utilization of different nanoparticles to control clearance, localization, and cell uptake and the chemical modification of the RNA molecule to make it more resilient to degradation and to increase its stability (20–23). The primary goal of this review is to investigate the role of ncRNAs, specifically miRNAs, lncRNAs, and circRNAs, as therapeutic targets and biomarkers in inflammatory and cancerous diseases. We aim to provide insights on their possible uses in non-invasive diagnostics, prognostics, and targeted therapies by examining their regulatory roles and mechanisms of action, highlighting recent developments, and identifying important research challenges and future perspectives. A thorough examination of existing literature was conducted following rigorous selection criteria to investigate the mechanistic role and therapeutic potential of ncRNAs in cancer and inflammatory diseases. Research articles, reviews, systematic reviews, and meta-analyses were sourced from reputable databases, including PubMed and Google Scholar. The search strategy employed keywords such as ncRNA, miRNA, lncRNA, circRNA, cancer, inflammation, autoimmune diseases, biomarkers, clinical trials, and therapy. Inclusion criteria required articles to be peer-reviewed and published between 2014 and 2025, except where older studies were necessary due to a lack of more recent information. Studies not published in English, conference abstracts, and those with inconclusive findings were excluded. By applying this methodology, we ensured that the presented findings were derived from high-quality and relevant sources, providing a comprehensive and up-to-date understanding of the topic.

## 2 Types of ncRNAs and their biogenesis

### 2.1 MicroRNAs (miRNAs)

**Figure 1:**
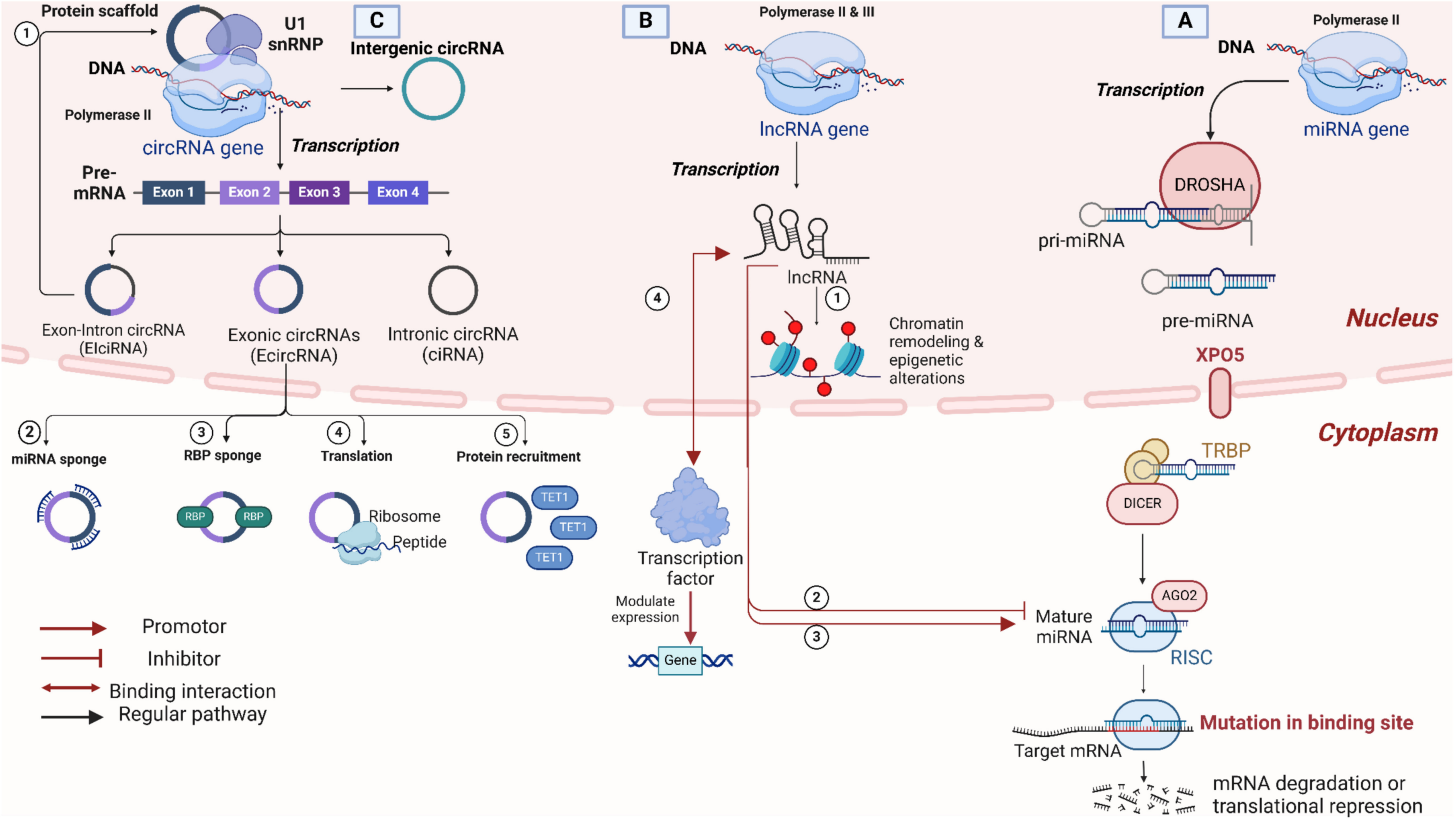
Overview of non-coding RNA types, biogenesis, and functional mechanisms. **(A)** MicroRNAs (miRNAs) Biogenesis and Function: miRNAs are transcribed by RNA Polymerase II and processed by DROSHA in the nucleus, forming precursor miRNAs (pre-miRNAs), which are exported to the cytoplasm by Exportin-5 (XPO5). DICER further processes pre-miRNAs in the cytoplasm to form mature miRNAs that associate with Argonaute (AGO2) within the RNA-induced silencing complex (RISC). miRNAs guide RISC to target mRNAs, leading to mRNA degradation or translational inhibition. **(B)** Long non-coding RNAs (lncRNAs) biogenesis and function: lncRNAs are transcribed by RNA Polymerase II or III. LncRNAs play critical roles in chromatin remodeling and epigenetic modifications in the nucleus. At the same time, in the cytoplasm, they regulate mRNAs and miRNAs by acting as enhancers, repressors, or binding transcription factors to influence gene expression. **(C)** Circular RNAs (circRNAs) biogenesis, types, and functions: circRNAs are generated through back-splicing, leading to a covalently closed loop structure that enhances stability. They can be classified as exon-intron circRNAs (EIciRNAs), exonic circRNAs (EcircRNAs), or intronic circRNAs (ciRNAs), each arising from different RNA regions. Key functional roles of circRNAs include acting as miRNA sponges, RNA-binding protein (RBP) sponges, templates for translation, and protein recruitment. Created in BioRender. Mohamed, A. (2025) https://BioRender.com/e46w484.

The biogenesis of miRNA starts from the nucleus, more precisely from sequences of DNA usually referred to as miRNA genes that are exclusively transcribed as miRNA transcripts, as shown in **Figure 1**. The final target of these miRNA transcripts can be the intronic or the untranslated region of a protein-coding gene. Previous studies have pointed to the canonical and non-canonical pathways as the major ones for illustrating the biogenesis of the miRNAs (1). Regarding the canonical pathway implied for the biogenesis of miRNA, the process initiates from the nucleus by transcribing the miRNA genes to give their relevant primary miRNAs with the help of polymerase III (24). The primary miRNAs comprise 60 to 80 nucleotides with a hairpin stem-loop structure. Following transcription comes the microprocessor complex that cleaves primary miRNAs to yield precursor miRNA (pre-miRNAs), which are around 22 base pair stems with 3′ 2 nucleotides that are overhang. The microprocessor complex is commonly referred to as “Drosha-DGCR8”, where Drosha acts as an RNase enzyme and DGCR8 is an RNA binding protein that stands for DiGeorge Syndrome Critical Region 8 (1, 2). The process then shifts from the nucleus to the cytoplasm with the contribution of Exportin 5, which exports pre-miRNAs to the cytoplasm to meet with the dicer. At this point, pre-miRNAs are processed by dicer, an RNase III endonuclease, which cleaves the hairpin structure to remove the terminal loop. This cleavage generates a short double-stranded RNA molecule known as the miRNA duplex. Then comes the big role of the RNA-induced silencing complex (RISC), which is assembled as a multiprotein complex comprising of the miRNA duplexes, Argonaute RISC Catalytic Component 2 (Ago2), and several other proteins all working together synergistically as a machine. Here, the miRNA duplexes are subjected to a final cleavage step by the Ago2, leaving a single-stranded miRNA that functions as a guide strand to scan mRNAs in the cytoplasm for potential complementarity, whereas the other strand gets degraded. In most cases, complementarity occurs in the 3’untranslated region (3’UTR) of mRNAs. Other less common complementarity cases have been reported, and they are found to be associated with not only the 5’UTR mRNA region but also the protein-coding ones (25). Finally, the guide strand reaches out to its target and is followed by either translation repression or degradation of the relevant protein (26). Other non-canonical miRNA biogenesis pathways have recently been revealed that are not dependent on either the microprocessor complex or dicer. To further illustrate, miRtrons does not include the microprocessor complex, as pri-miRNAs are spliced instead. Other scenarios emerge that happen to surpass the dicer and are routed directly to Ago2, in which case the stem-loop is too short to be recognized by the dicer (1).

### 2.2 Long non-coding RNAs (lncRNAs)

lncRNAs, unlike miRNA, are long RNA transcripts found to exceed 200 nucleotides that do not get translated into protein (non-coding transcripts). The biogenesis process is like that of miRNA, but it has some distinctive characteristics. First of all, both RNA polymerase II and III can initiate the transcription process depending on the associated promoter sequence, as shown in **Figure 1** (27). lncRNAs are further clustered according to their gene location, which is different from protein-coding ones. Grouping scenarios involve inter or intra-genic lncRNAs. The latter is identified as sense or antisense based on the orientation associated with the overlap condition. There are also intronic or overlapped ones with genes that code for proteins (28). The transcription process involved in lncRNAs is mostly under the control of enhancers or promoters to regulate its function. Following transcription, transcripts are processed by splicing, capping, and poly-A tail addition. Not to mention, the transcripts generated affect the expression of other genes. As a result, the majority of lncRNAs serve as regulatory elements, with a subset of lncRNAs being converted into miRNAs or piRNAs, which also play regulatory roles. Interestingly, the function of the lncRNAs dictates the place it localizes, where it is either directed to the nucleus or the cytoplasm. To elaborate, localization directed to the nucleus is typically involved in the process of chromatin-modifying complexes. In contrast, those directed to the cytoplasm are involved in the interaction with not only mRNA but also proteins as a part of the post-transcriptional regulation (3). As an example of nuclear localization, chromatin remodeling is portrayed in the role of XIST lncRNA to inactivate the X-chromosome by alterations in the gene expression of genes associated with X chromosomes (29). Another type of nuclear control is epigenetic regulation, which occurs when lncRNAs interact with histone proteins, methyl transferase enzymes, and transcription factors to activate or otherwise repress gene expression. Thus, lncRNAs can also have a role in the transcription process by working as a repressor or enhancer in collaboration with transcription factors or other RNA-binding proteins (30). Cytoplasmic localization, on the other hand, is concerned with post-transcriptional regulation, involving the interaction of lncRNAs with either mRNA, altering its stability, or with miRNA, inhibiting their translational repression role of target mRNA (31).

### 2.3 Circular RNAs (circRNAs)

Concerning circRNAs, non-canonical splicing drives the formation of circRNAs in human cells by the back-splicing mechanism, resulting in a closed-loop structure held by a covalent bond, as illustrated in **Figure 1**. Such circular structure has several add-ons compared to linear RNAs, including excellent stability and resistance to exo-nuclease degradative activity (4).,When it comes to the biogenesis process, circRNAs follow two distinguished pathways: spliceosome-mediated back-splicing and lariat-driven circularization. Regarding the spliceosome-mediated back-splicing, exons forming the pre-mRNA are joined in a way that the 5’end of the final exon undergoes back-splicing to an initial exon’s 3’ end, forming the circular structure. This gives a covalently bonded circular loop made up entirely of exons, and no introns are included in such a pathway, resulting in enhanced stability (32, 33). The lariat-driven circularization, on the other hand, includes both exons and introns and hence is stable but less stable than the other pathway due to the presence of introns. The lariat structure is seen during the normal splicing process in case the 5’ end of an intron is connected to a branching point inside the intronic area. The lariat’s 3’ end connects back to its 5’ as an alternative to degradation, forming a circular structure (34, 35). circRNAs function in several processes on the cellular level, where they work as a sponge for miRNAs to regulate gene expression (36). circRNAs are not simply regulatory elements; they have also been proven to interact with other proteins and translate into proteins (37).

## 3 ncRNAs in cancer

Genetic and epigenetic changes in normal cells drive the development of cancer characteristics, which in turn cause malignancies to develop and spread (5). Even though protein-coding genes have traditionally been the focus of cancer research, the finding that non-protein-coding sequences make up approximately 97% of the human genome has directed attention toward studying this genetic matter in tumorigenesis (8, 38, 39). Abnormal expression of ncRNA, along with the downstream signaling processes, including signal transduction, chromatin remodeling, and transcriptional and post-transcriptional modification, affect the onset and progression of various human cancers (40). ncRNA acts as oncogenes or tumor suppressors to form complex networks of mutual interactions and modulate several signaling pathways (6). They also display tissue-specific expression patterns, which are frequently dysregulated in cancer. As a result, ncRNAs become promising targets for diagnosis, prognosis, and treatment (8).

### 3.1 Role of miRNA in cancer

Abnormal expression of miRNA is observed in nearly all forms of human cancer, where they can significantly interfere with cell signaling pathways, affecting the onset and progression of cancer (41). Specific regulatory functions of miRNAs reveal modulation of important gene expression linked to the cancer cell cycle, apoptosis, and migration (42). The impact of miRNAs on cancer can primarily be observed in three ways: as tumor suppressors, as oncogenes, and by either promoting or inhibiting cancer metastasis (43). In chronic B-cell lymphocytic leukemia (B-CLL), miR-29 functions as a tumor suppressor that inhibits cancer progression; yet, it is also enhanced in acute myeloid leukemia and more aggressive types of B-CLL, suggesting that it may also have an oncogenic role (39). Certain miRNAs can prevent normal cells from transforming into cancerous ones. Their expression is often downregulated in cancer compared to normal cells (44). For example, let-7 is a tumor suppressor usually downregulated in cervical, breast, and lung cancers. Let-7 inhibits the RAS family associated with approximately one-third of human cancers (45, 46). Similarly, miR-15a/miR-16-1 is downregulated in CLL (47). The miR-34 family, which consists of the three members miR-34a, miR-34b, and miR-34c, is the first family of known tumor suppressors that works together with the tumor suppressor gene p53 (48–50). Both miR-34a and miR-34b can inhibit cancer cell invasion and metastasis, whereas miR-34a is downregulated in rectal cancer (51, 52). Additionally, miR-34 negatively regulates the Wnt signaling pathway, leading to an inhibition of the epithelial-mesenchymal transition (EMT) process, which is linked to invasion and migration. miR-34 is found to have lower expression levels in prostate cancer and downregulated in multiple myeloma, osteosarcoma, breast, and gastrointestinal cancers (39, 50, 53).

When acting as oncogenes, miRNAs are upregulated in cancer cells to promote proliferation and metastasis or inhibit tumor suppressor activities (54). For instance, miR-155 contributes to lymphoma development by facilitating abnormal B cell proliferation (55). Another example is miR-21, which functions as an oncogenic miRNA by downregulating tumor suppressor genes, hence contributing to tumorigenesis (56). miR-21 is upregulated across numerous cancer types, and its overexpression induces pre-B-cell lymphoma in murine models and activates the Ras/MEK/ERK signaling pathway, leading to KRAS-dependent carcinogenesis (57, 58). Also, miR-17-92 cistron is upregulated in several lymphoma types and collaborates with the oncogenic factor c-myc to enhance cancer development (59). Additionally, LATS2, a tumor suppressor gene, is downregulated by miR-372 and miR-373 to balance wild-type TP53, consequently interacting with RAS and promoting cellular proliferation and tumorigenesis as part of the mechanism underlying human testicular germ cell tumorigenesis (39). RAS mutations are substantial contributors to cancer development, with altered RAS genes allowing the RAS protein to remain active, thereby regulating cancer cell proliferation, metabolism, apoptosis, and angiogenesis through downstream MAPK, PI3K, and other signaling pathways (60, 61). Moreover, non-canonical miRNAs are found to be associated with tumor formation and progression of the disease in major cancer types. An example is pre-miR-451, which is downregulated in cancer of the epithelial cells of different organs involving the lung and colon (1).

Besides their direct roles in promoting or inhibiting cancer, miRNAs can also influence cancer metastasis by promoting the growth and expansion of the primary tumor, invasion of surrounding tissues, and penetration into lymphatic and blood vessels (62, 63). Additionally, tumor thrombi may migrate through lymph and blood, ultimately extravasating and invading distant tissues, where cancer cells can proliferate and establish metastasis (64). Research has identified several miRNAs that promote cancer metastasis. For example, the upregulation of miR-10b enhances breast cancer metastasis (65). Additionally, induction of exosomal miR-21 and miR-10b by the acidic TME of the liver can promote both liver cancer cell proliferation and metastasis (39, 66). Moreover, miRNAs have been shown to play a role in inhibiting cancer metastasis. For instance, miR-335 directly inhibits the production of SOX4, a transcription factor involved in the development and migration of cellular progenitors, therefore preventing breast cancer metastasis (67). Additionally, miR-126 targets CRK37, a protein involved in actin remodeling and an adaptor in signaling for focal adhesion formation and cell migration, and can hence suppress cancer cell adhesion, migration, and invasion (39, 68). Furthermore, miR-146a downregulates Rock1 expression, which is significant for morphogenesis and hyaluronan-mediated transformation and metastasis of hormone-refractory prostate cancer *in vivo* (39). Therefore, miRNAs have the potential to serve as biomarkers for differentiating metastatic and non-metastatic cancers (41, 69, 70). For example, miR-10b can effectively differentiate between metastatic melanoma and its non-metastatic counterpart (39).

miRNAs also play a significant role in modulating alternative splicing (AS) by targeting splicing factors (SFs) or RNA-binding proteins (RBPs) in cancer (71). For example, miR-133b inhibits SF3B4 mRNA translation, leading to a disruption of SF3B4-regulated AS and, limitation in hepatocellular carcinoma (HCC) cell proliferation and metastasis (72). Similarly, miR-200c and miR-375 exert translational repression on Quaking (QKI), an RBP, which affects QKI-mediated AS and consequently influences the plasticity of cancer-associated epithelial cells (71). Furthermore, the miR-212/hnRNPH1 axis plays a role in prostate tumorigenesis by downregulating the expression of the androgen receptor (AR) and its splice variant AR3 (73). Specific miRNAs, including miR-21, miR-106a, miR-126, miR-155, miR-182, miR-210, and miR-424, are critical regulators of tumor angiogenesis (74). miR-21, miR-126, and miR-93 enhance the expression of vascular endothelial growth factor (VEGF) and hypoxia-inducible factor 1 (HIF-1) by targeting phosphatase and tensin homolog (PTEN) and inhibiting the angiogenesis inhibitor thrombospondin-1 (THBS1) (75, 76). Additionally, miRNAs can interact with lncRNAs, such as MALAT1, to inhibit large tumor suppressor 2 (LATS2), thereby regulating cancer cell growth, invasion, and metastasis (74, 77).

Cancer development is well characterized by abnormal glucose metabolism. Typically, normal tissues depend on aerobic oxidative phosphorylation to generate energy but switch to anaerobic glycolysis during hypoxic conditions. In contrast, cancer cells predominantly rely on aerobic glycolysis, even with sufficient oxygen (78–80). Numerous ncRNAs involved in glucose metabolism are dysregulated in various cancers. This dysregulation can lead to mitochondrial dysfunction, enhanced activity of key enzymes, altered isozyme profiles, and disrupted glucose metabolic signaling pathways (81). Regarding ncRNAs and glucose uptake, GLUT is a crucial transporter of glucose —the abnormal expression of GLUT1 results from the dysregulation of ncRNAs in cancer, including miR-181a and let-7a-5p. Increased expression of miR-181a has been associated with increased metastasis in colorectal cancer (CRC) by inhibiting PTEN expression while enhancing GLUT1 expression. Conversely, low levels of let-7a-5p in triple-negative breast cancer (TNBC) correlate negatively with GLUT12 and promote the proliferation, migration, and invasion of TNBC cells (81, 82). More examples of different miRNAs and their role in various types of cancer are summarized in **Table 1**.

**Table 1:** Overview of non-coding RNAs (ncRNAs) involved in cancer: expression profiles, target pathways, and associated cancer types.

ncRNA Type	Name	Expression	Target Pathway/Gene	Cancer Type	References
miRNA	miR-15a-3p	Downregulated	HMOX1, CDC37L1, PPIA	Hepatocellular carcinoma	(299, 300)
	miR-101		EZH2, H3K27me3		(301)
	miR-223		mTOR pathway, hypoxia-inducible factor 1α-driven CD39/CD73-adenosine pathway, Rab1, NLRP3		(302, 303)
	miR-122		Bcl-2 family proteins, Wnt/β-catenin-TCT signaling pathway, SUV39H1, histone H3K9 methylation, acetylation		(304–306)
	miR-1		FoxP1, MET, HDAC4		(306)
	miR-191	Upregulated	has_circ_0000204/miR-191/KLF6 axis, EMT		(66, 307)
	miR-519d		AMPK Signaling Pathway, Rab10, CDKN1A/p21		(308)
	miR-429		PTEN, EMT, PD-L1		(306, 309)
	miR-148a	Downregulated	DNMT1, NF-kB		(306, 310)
	miR-155	Upregulated	STAT3, c-MYC, PIK3R1-PDK1/AKT-FOXO3a pathway	Breast Cancer	(311, 312)
	miR-34a		LDHA		(311)
	miR-195		ACC, Lipid Homeostasis		
	miR-21	Upregulated	PTEN, STAT3	Multiple cancers	(74, 208)
	miR-223		Notch1, EMT	Pancreatic cancer	(208)
	miR-301a		PTEN, P63, TAp63		
	miR-429		PDCD4		
	miR-181a		GLUT1, PTEN	Colorectal cancer	(81)
	let-7a-5p	Downregulated	GLUT12	Triple-negative breast cancer	
	miR-29c		VEGFA	Lung adenocarcinoma	(313)
	miR-145-5p		CP		
	miR-519c		HIF-1α±/VEGFA axis	Non-small cell lung cancer	
	miR-497		HDGF, VEGFA		
	miR-619-5p	Upregulated	RCAN1.4		
	miR-141		KLF12, GAX	Small and non-small cell lung cancer	
	miR-520d-5p	Downregulated	PTK2	Cervical cancer	(314)
	miR-214		EZH2		
	miR-31	Upregulated	BAP1		
	miR-138	Downregulated	SIRT1		
	miR-675	Upregulated	RUNX1	Gastric cancer	(315)
	miR-214	Downregulated	DNMT1, TP53	Testicular cancer	(316)
	miR-320a/383		NLC1-C (narcolepsy candidate-region 1 genes)		
	miR-17/92	Upregulated	MYC, PTEN, TP53INP1	Chronic lymphocytic leukemia	(317)
lncRNA	MIAT	Upregulated	OCT4		
	DLEU1, DLEU2	Downregulated	NF-kB		
	NEAT1	Upregulated	p53		
	SPRY4-IT1		MAPK/ERK, PI3K/Akt	Testicular cancer	(316)
	HOTTIP		miR-1283p/HOXA13		
	ZFAS1		HMGA2	Papillary thyroid cancer	(318)
	AB074169	Downregulated	KHSRP, CDKN1a		
	LINC00173 V1	Upregulated	miR-511-5p/VEGFR axis	Lung squamous cell	(313)
	MCM3AP-AS1		miR-340-5p/KPNA4 axis	Non-small cell lung cancer	
	MCM3AP-AS1		miR-194-5p/FOXA1	Hepatocellular carcinoma	(319)
	MIAT		Histone acetylation		(320)
	SRHC	Downregulated	Epigenetic silencing		(321)
	FENDRR		miR-423-5p/GADD45B, glypican-3		(9, 322–330)
	TUG1	Upregulated	miR-144-3p, miR-382/EZH2, SOX2, MYC		(331, 332)
	MINCR		c-Myc/CDK2, CDK4	Non-small cell lung cancer, hepatocellular carcinoma, and gallbladder cancer	(333)
	PVT1		miR inactivation	Cervical cancer	(334)
	SNHG16		PI3K/AKT pathway/ miR-373/EGFR axis	Glioma	(335)
	HOTAIR		TGF-β, PI3K/AKT/MAPK, Wnt/β-catenin	Breast and liver cancer	(86)
	FAL1		PRC1	Ovarian cancer	(38)
	SNHG15		miR-345-5p	Breast cancer	(336)
	SNHG7		c-Myc, LDHA		(311)
	NEAT1		miR-506, STAT3	Triple-negative breast cancer	
	H19		Wnt/β-catenin, PI3K-Akt-mTOR, miR-675, let-7/HIF-1α/PDK1, EZH2	Various cancers	(311, 315, 316, 331, 337)
	ZEB2-AS1		EMT/ E-cadherin		(338, 339)
	MEG3	Downregulated	P53, autophagy, miR-21-5p/PTEN axis, TGFBR1/SMAD2		(39, 332, 340–342)
	MALAT1	Upregulated	G2/M transition, LATS2, SRSF1 via the Wnt pathway/c-Myc axis, splicing factor Myb,		(39, 74, 77, 102, 103, 331)
	HULC		miR-613, vimentin	Colon cancer	(343)
	CCAT1		c-MYC, PI3K/AKT	Colorectal cancer	(27, 112)
	HOTAIR		VEGF, angiogenesis, PRC2 complex (Histone methylation)	Gynecological cancers	(332, 344)
	XIST		Tumor progression, EMT	Multiple myeloma	(345)
	linc-ROR		miR-145/Nanog	Pancreatic Cancer	(331)
	LINC00668		VEGF, miR-297, proliferation	Oral cancer	(346)
circRNA	circPRKCI	Upregulated	AKT signaling		
	circ_0006988		miR-491-5p/MAP3K3 axis	Non-small cell lung cancer	(313)
	circ_0016760		miR-29b/HIF1α ± axis		
	circLMO1	Downregulated	miR-4192/ACSL4	Cervical Cancer	(314)
	circAKT3	Upregulated	miR-198, PIK3R1, cisplatin resistance	Gastric cancer	(347)
	circPDSS1		miR-186-5p, promotes cell cycle and proliferation		
	circHECTD1		miR-1256, promotes glutaminolysis, autophagy		
	circYY1		miR-769-3p	Breast Cancer	(311)
	circSAMD4A		PFKFB3, chemoresistance	Colorectal cancer	(81)
	circITGA7		ITGA7		(240)
	circTRIM33-12	Downregulated	miR-191	Hepatocellular carcinoma	(348)
	circSOD2	Upregulated	JAK2/STAT3 signaling pathway, EP300, WDR5, H3K27ac, H3K4me3		(349)
	circMET		miR-30-5p/Snail/DPP4/CXCL10		(306)

### 3.2 Role of lncRNA in cancer

Many lncRNAs exhibit tumor-related cell- or tissue-specific expressions, indicating their potential role as therapeutic targets. lncRNAs affect gene expression within the nucleus by regulating epigenetic and transcriptional mechanisms and in the cytoplasm by modifying post-transcriptional and translational processes (27, 83, 84). Like miRNAs, lncRNAs can function as oncogenes and tumor suppressors (85). For instance, overexpression of lncRNA HOTAIR has been implicated in the progression of various cancers (86, 87). Elevated HOTAIR levels can inhibit the activity of tumor suppressors such as protocadherin 10 (PCDH10), progesterone receptor (PGR), protocadherin β5 (PCDHB5), and junctional adhesion molecule 2 (JAM2), leading to tumorigenesis (39). It also has a distinguished role associated with immune evasion in the TME (88). H19, the first identified lncRNA overexpressed in HCC and rectal cancer, influences cancer development. H19 serves as a precursor for miR-675, and its elevated expression correlates with an upsurge in miR-675 levels, contributing to rectal cancer progression (89–91). lncRNAs can also function as tumor suppressors. TERRA, categorized as telomeric repeat-containing RNAs, are a group of lncRNAs transcribed from telomeres that may negatively regulate telomerase activity, thereby acting as tumor suppressors (39). MEG3 exhibits downregulation in cancer cells. In bladder cancer, the overexpression of MEG3 induces autophagy and contributes to p53 accumulation (92). LINC01089, for instance, is an oncogenic lncRNA that functions in liver cancer by stimulating the invasion of cancerous cells by its effects on the ERK/Elk1/Snail axis involved in the signaling pathway. LINC01089 is found to be a super-enhancer lncRNA consisting of a set of enhancers with many transcription factors that stimulate the transcription of oncogenic genes. Hence, targeting the super-enhancer can be a very promising, novel, and successful approach to cancer treatment (93).

lncRNAs interact with RBPs to regulate target genes, which can have a significant impact on AS (H. Huang et al., 2021). For instance, esophageal squamous cell carcinoma (ESCC) is associated with a poor prognosis when the lncRNA DGCR5 is highly expressed. *In vitro*, DGCR5 silencing decreases ESCC cell migration, invasion, and proliferation (94). AS is frequently associated with transcription processes in terms of transcriptional regulation, and lncRNAs can also affect AS through this mechanism (27, 71, 95). As an example, the lncRNA PVT1b inhibits the transcriptional activity of c-Myc, which reduces the proliferation of lung and pancreatic cancer cells (96–98). The expression of heterogeneous nuclear ribonucleoprotein (hnRNP) proteins, which dysregulate pyruvate kinase mRNA splicing in cancer, is known to be facilitated by the oncogenic transcription factor c-Myc (99–101). Additionally, HCC is affected by the oncogenic effects of the upregulated lncRNA MALAT1 (102). Through the Wnt pathway and c-Myc axis, MALAT1 transcriptionally activates the oncogenic splicing factor SRSF1. MALAT1 can modulate B-MYB AS and aid in the progression of the cell cycle by acting as a splicing factor decoy (71, 103).

While most lncRNAs are thought to be non-coding, some lncRNAs have been found to have translational effects and encode short peptides in certain situations (71). For instance, the conserved 53-amino acid peptide produced by the lncRNA HOXB-AS3 is associated with a poor prognosis in patients with CRC. The RNA-binding protein hnRNPA1 and the CRC growth are both inhibited by the HOXB-AS3 peptide, which also modifies the AS of pyruvate kinase M (PKM). Due to this modulation, the PKM2 isoform is formed less frequently, which ultimately helps suppress the growth of CRC (104–106). Additionally, it has been demonstrated that lncRNA LOC90024, which encodes the small peptide Splicing Regulatory Small Protein (SRSP), promotes the development and metastasis of CRC. To affect AS, SRSP interacts with several splicing regulators, including SRSF3. SRSP increases SRSF3’s binding to exon 3 of the transcription factor Sp4, which results in the production of the oncogenic isoform Sp4-L and the inhibition of the non-oncogenic isoform Sp4-S (107–111). Another example is the nuclear lncRNA CCAT 1, which interacts with the CCCTC-binding transcription factor (CTCF) to function as an oncogene and control the intra-chromosomal interactions of known oncoproteins like c-MYC, thereby promoting CRC tumorigenesis (27, 112). More examples of different lncRNAs and their role in various types of cancer are summarized in **Table 1**.

### 3.3 Role of cricRNA in cancer

circRNA can influence glucose metabolism by modulating some key enzymes (81). For instance, To prevent cell apoptosis, circ-Amotl1 translocates to the nucleus and interacts with PDK1 and AKT1 (113–115). Cichipk3 can sponge miR-124, which then inhibits the expression of several transporters and glycolytic enzymes (115, 116). Moreover, circRNF13 is essential for the programming of nasopharyngeal carcinoma (NPC) glucose metabolism because it stabilizes SUMO2 mRNA, which increases GLUT1 ubiquitination and SUMO degradation (81, 117). In CRC, the upregulation of lncRNA GAL promotes liver metastasis. GAL interacts with GLUT1 to improve SUMOylation, which increases GLUT1 mRNA stability (81, 118). Furthermore, by sponging miR-760, circDENND4C increases GLUT1 expression, which increases the growth of CRC (119–121). m6A modification of circFOXK2 increases GLUT1 stability, which subsequently plays a carcinogenic role in oral squamous cell carcinoma (OSCC) (122). Furthermore, circRNAs affect lipid metabolism. Abnormalities in lipid metabolism can influence cancer development, with increased lipolysis contributing to cancer cachexia, a syndrome characterized by significant fat loss (123–126). For example, circ-0046367 binds to miR-34a, protecting peroxisome proliferator-activated receptor (PPAR)α from transcriptional repression. Lipid degradation is stimulated through activation of CPT2 and ACBD3 by PPARα (39).

circRNA regulates metabolism, but it can also affect transcription, mRNA splicing, protein localization, and activity through interactions with RNA polymerase II and small nuclear RNA (snRNA) (37, 127). Higher expression levels of circRNA FBXW7 correlate with lower patient survival rates compared to those with lower expression, and the FBXW7-185AA protein, encoded by glioblastoma circRNA FBXW7, plays a critical role in glioblastoma formation and prognosis, its expression is reduced in glial tissue (39, 128). The functional protein SHPRH-146aa, which is encoded by circ-SHPRH, functions as a tumor suppressor in glioblastoma and is expressed at a reduced level in this type of cancer (129, 130). Additionally, circURI1 functions as a tumor suppressor, while circRNA_0000285, circRNA WHSC1, and hsa_circ_001783 are recognized as oncogenes (39).

Through a variety of mechanisms, such as functioning as miRNA sponges, interacting with RBPs, controlling transcription, and acting as translation templates, circRNAs play crucial roles in tumorigenesis (71). One AS modulator implicated in the development and metastasis of cancer is circURI1. circRNA profiling of five paired gastric cancer (GC) and nearby non-cancerous tissue specimens revealed the presence of circURI1. circURI1 promotes GC metastasis *in vitro* and *in vivo* and shows higher expression levels in GC compared to para-cancerous tissues (131, 132). Their function as miRNA sponges or competing endogenous RNAs (ceRNAs) is one common way that circRNAs function. circRNAs may be linked to AS when their targets are SFs (133–135). By altering the expression of epithelial splicing regulatory protein 1 (ESRP1) through the miR-526b-5p/c-Myc/TGF-β1 pathway, circUHRF1 promotes OSCC (136–138). CiRS-7, which acts as a sponge for miR-7, contributes to the advancement of various malignancies, including CRC, via interacting with the MAPK and PI3K pathways (139, 140). circ-LRIG3 is another circRNA that helps promote metastasis in HCC by upregulating the MAPK pathway (141). More examples of different circRNAs and their role in different types of cancer are summarized in **Table 1**.

## 4 ncRNAs in inflammatory diseases

ncRNAs have been identified as being important in a variety of diseases, especially inflammatory ones. Sepsis and autoimmune diseases can result from the dysregulation of inflammation, even though it is a protective response (142). ncRNAs regulate gene expression and influence differentiation, proliferation, and cell survival, which are key processes in inflammation (143–146).

### 4.1 Role of miRNAs in inflammation

miRNAs influence inflammation from initiation to resolution through positive and negative feedback. Positive feedback aids in pathogen defense and tissue repair, while negative feedback maintains tissue homeostasis during severe inflammation (147). An example would be miR-155, a miRNA derived from the ncRNA transcript of the B cell integration cluster (BIC) protooncogene (148). miR-155 is conserved in humans and mice (149). It is induced by transcription factors such as AP-1, MYB, PU.1, STAT3, and Ets2, particularly through AP-1 during toll-like receptor (TLR) agonist activation. It targets several inflammatory mediators, including TNF-α and interferons. In contrast, the anti-inflammatory cytokine IL-10 downregulates miR-155 by suppressing TLR4 signaling and inhibiting Ets2 transcription in a STAT3-dependent manner in response to lipopolysaccharide (LPS). miR-155 is highly expressed in various innate and adaptive immune cells, including monocytes and macrophages. It promotes macrophage proliferation and pro-inflammatory cytokine secretion (IL-6 and TNF-α) by targeting SHIP1 and SOCS1. Additionally, SOCS1, a target of miR-155, negatively regulates M1 macrophage polarization by inhibiting the JAK2/STAT1 pathway and TLR/NF-κB signaling. SOCS1, targeted by miR-155, inhibits M1 macrophage polarization by suppressing the JAK2/STAT1 and TLR/NF-κB pathways (150, 151). Evidence shows that miR-155-3p is also active in regulating different genes. miR-155 has confirmed targets that can induce both pro- and anti-inflammatory responses, including negative regulators like SOCS1 and SHIP-1, which, when suppressed, promote inflammation (152). Notably, miR-155 can have anti-inflammatory effects as well; for example, it targets PI3Kγ to suppress mast cell activation. In LPS-activated macrophages, miR-155 inhibits TLR signaling by targeting IRAK1 and TRAF, creating a negative feedback loop that reduces macrophage activation. However, overexpression of miR-155 can paradoxically increase LPS-induced TNF production in both *in vitro* and *in vivo* settings (153). Systemic lupus erythematosus (SLE) is an autoimmune disorder with reduced and impaired regulatory T cells (Tregs) and is associated with higher cancer rates and mortality (154). A study was conducted to investigate the role of miR-155 and SOCS1 in Treg function and stability in SLE. SLE patient and SLE-induced mouse Tregs showed decreased SOCS1 and increased miR-155 levels. Inflammatory cytokine-stimulated Tregs required NF-κB signaling for altering SOCS1 and miR-155 expression. miR-155 negatively regulates SOCS1, diminishing Treg function and differentiation. Conversely, inhibiting miR-155 enhances Treg function and improves SLE conditions in mice. Inflammation-driven miR-155 compromises Treg stability by lowering SOCS1, suggesting that targeting miR-155 could be a potential SLE treatment (155).

Unlike miR-155, some miRNAs have a dual role in inflammatory response. This includes miR-21, which acts both as a pro-inflammatory agent and as an inhibitor of inflammation. It is associated with various illnesses, particularly malignancies, and functions as a key regulator of inflammatory processes. Studies show miR-21 is upregulated in asthmatic children and experimental asthma models while downregulated in cord blood monocytes from children with allergic rhinitis, linking it to airway inflammatory diseases. Increased miR-21 levels are linked to conditions like asthma and lumbar spinal canal stenosis, which promotes inflammation (156). In addition to that, alterations in the expression of miR-21-5p downregulate TLR/NF-κB signaling by reducing TRAF6 and PDCD4 levels, leading to decreased proinflammatory cytokines in certain types of cells such as human dental pulp cells (hDPCs) (157). Evidence shows that miR-21-5p is crucial in TGF-b1 signaling in keratinocytes (KCs). TGF-b1 increases miR-21-5p expression via transcriptional and post-transcriptional mechanisms involving SMADs and RNA helicase p68. Overexpression of miR-21-5p boosts KC proliferation and migration by targeting PTEN, PDCD4, and TIMP3 while inhibiting the G1-to-S phase transition through CDC25A downregulation and CDK2 activation. Additionally, miR-21-5p is linked to skin inflammation, being highly expressed in psoriatic lesions, where its upregulation affects the IL-6/STAT3 pathway and activates TACE/ADAM17. IL-22 also induces miR-21-5p, which, alongside miR-21-3p, promotes KC proliferation via cyclin D1 upregulation (158). In addition to miR-21-5p, miR-31 potentially induces wound edge keratinocytes and is regulated by NF-κB and STAT3 during inflammation. Using miR-31 loss-of-function mouse models, a study by Shi et al., 2018, demonstrated that miR-31 enhances keratinocyte proliferation and migration by activating the Ras/MAPK pathway by targeting negative regulators Rasa1, Spred1, Spred2, and Spry4 (159).

Furthermore, some miRNAs regulate inflammation, hematopoiesis, allergic responses, and aspects of the innate immune system. For example, miRNA-146a is a single-stranded ncRNA that serves as a diagnostic and prognostic biomarker for various diseases. Polymorphisms in miRNA-146a can influence the development of autoimmune disorders and cancer. It affects gene expression by controlling proteins like IRAK1, IRAK2, and TNF receptor 6 and modulates pathways such as TNF, NF-κB, MEK-1/2, and JNK-1/2. Research shows that miRNA-146a’s role in stem cell synthesis contributes to tumorigenesis (160). miRNA-146a targets IRAK1 and TRAF6, proteins involved in TLR and IL-1 receptor signaling, to inhibit proinflammatory mediator secretion and block TLR signals. miRNA-146a is crucial for endotoxin-induced tolerance, reducing monocyte responsiveness to LPS after repeated exposure and preventing inflammation. LPS exposure raises miRNA-146a levels in THP-1 cells, negatively correlating with TNF levels, indicating LPS tolerance (161). Positive regulation of miRNA-146a is essential for tolerance activation; its transfection induces tolerance without LPS priming, while its knockdown diminishes LPS tolerance (162). miRNA-146a is a key negative feedback regulator in vertebrate innate immunity, primarily targeting components of the MyD88-dependent signaling pathway, reducing inflammatory mediator synthesis. While research has mainly centered on the TLR pathway and inflammatory cells, miRNA-146a is also expressed in various cell types and may have other functions depending on the context (160, 163).

miR-122 plays a role in immune response and enhances p65-mediated NF-κB signaling by targeting IκBα. The NF-κB p65 subunit’s NLS interacts with IκBα’s ANK1, while the p50 subunit’s NLS connects with IκBα’s ANK2, forming a trimer that sequesters NF-κB in the cytoplasm (164). A study was conducted on endothelial progenitor cells (EPC cells) co-transfected with NF-κB, IL-1β or IL-8 reporters, and IκBα shows that the overexpression of p65-activated these reporters, whereas IκBα inhibits this activation. miR-122 down-regulates IκBα, promoting the activation of NF-κB, IL-1β, and IL-8 reporters. Luciferase assays confirmed that miR-122 facilitates this activation, and its inhibitor reversed miR-122’s effects. This suggests that miR-122 exerts an inhibitory effect on the levels of IκBα mRNA and may facilitate the degradation of IκBα mRNA. This indicates that miR-122 plays a regulatory role in modulating IκBα and, consequently, the innate immune response (165).

miR-26a expression was reported to rapidly decrease after TLR4 stimulation in microglia. Overexpression of miR-26a significantly lowered inflammatory cytokines like TNF-α and IL-6, while its knockdown increased their production. miR-26a negatively regulates LPS-induced cytokine production in microglia, partly by targeting ATF2 (166). Furthermore, a previous study indicates that miR-26a overexpression disrupts PI3K signaling, which is a pathway involved with cancer and therapy, primarily targeting PTEN to impede early B cell development (167). An inverse relationship between miR-26 levels and IL-6 has been observed in some tumor cells, suggesting that miR-26 may regulate inflammation and tumorigenicity by down-regulating IL-6. IL-6, a multifunctional cytokine, plays significant roles in chronic inflammatory diseases and promotes tumor cell proliferation, survival, angiogenesis, and immune tolerance (168). Previous studies indicate that miR-124 regulates microglial polarization, promoting the transition from pro-inflammatory M1 to anti-inflammatory M2. It reduces inflammation by downregulating TNF-α, major histocompatibility complex II, and reactive oxygen species. Furthermore, miR-124 is a key regulator of microglial quiescence in the central nervous system (CNS) and a novel modulator of monocyte and macrophage activation (169).

### 4.2 Role of lnRNAs in inflammation

lncRNAs are major players in the inflammatory pathway as they have crucial roles in not only expression but also differentiation of cytokines and immune cells, respectively (170). The transcription of inflammatory genes is influenced by lncRNAs, which are important regulators of the inflammatory response. Their functions include enhancing or suppressing inflammatory transcription, modulating gene-specific, time-dependent epigenetic control of inflammatory transcription, and acting as scaffolds for RNA-binding proteins in chromatin remodeling (171). TLR2 activation triggers the production of several lncRNAs, such as THRIL (TNFα- and hnRNPL-related immunoregulatory lncRNA), which can induce inflammatory responses through signals such as NF-κB signaling (172). THRIL knockdown decreases TNF-α secretion in unstimulated cells and following TLR2 activation. Through its interaction with hnRNPL, THRIL acts as a scaffold, forming a complex that binds to the TNF-α promoter to increase transcription. PACER is upregulated and controls the expression of the neighboring inflammatory gene PTGS2 (COX-2) after stimulation by LPS, IL-1β, or TNF-α (171, 173, 174). Furthermore, MALAT1 influences T-cell function, with naïve T-cell activation leading to reduced expression (175). The conserved protein MALAT1 is expressed in septic rat hearts, cardiac microvascular endothelial cells, and LPS-activated macrophages. It is also linked to several human cancers and immune responses, especially acute inflammation (176, 177).

By modifying several immune genes, lincRNA-Cox2, a lncRNA that is located next to the Ptgs2 (Cox2) gene, is also known to be an essential regulator of inflammatory responses. Like Ptgs2 induction via the MyD88 and NF-κB pathways, LPS stimulation increases lincRNA-Cox2 expression in dendritic cells and bone marrow-derived macrophages (BMDM). In BMDM, lincRNA-Cox2 overexpression or silencing affects important immune genes such as TNFα, IL-6, CCL5, SOCS3, and STAT3. Among the mechanisms of action are cytosolic IKB-α degradation, binding with hnRNP A/B and A2/B1, participation in the SWI/SNF complex, increasing NF-κB activity, and chromatin remodeling. It was recently demonstrated that lincRNA-Cox2 increases the transcription of TNFα-induced IL-12b by attracting the Mi-2/NuRD repressor complex to its promoter. Overall, acute inflammatory signaling is significantly influenced by lincRNA-Cox2 activation (178–182). Another example is lincRNA-p21, which was first discovered to be a repressor of p53-dependent transcription. The expression of many p53-repressed genes changed when lincRNA-p21 was silenced; these changes could be undone by blocking p53, suggesting that lincRNA-p21 functions as a downstream repressor of p53. Interaction with hnRNP-K is a part of this repression mechanism. The positive correlation between p53 levels and lincRNA-p21 expression in rheumatoid arthritis (RA) patients raises the possibility that basal lincRNA-p21 levels in peripheral blood mononuclear cells (PBMCs) are p53-independent (180, 183).

lncRNA HOTAIR is crucial for cytokine regulation and inflammation in macrophages. During LPS-induced inflammation, HOTAIR is upregulated in macrophages, where it controls NF-κB activation by influencing IκBα degradation. This affects the expression of pro-inflammatory and cytokine genes, such as IL-6 and iNOS. GLUT1 is expressed in macrophages in response to LPS, and it was found that HOTAIR controls this expression, affecting glucose uptake and metabolism, which is essential for the cells’ energy during inflammation. As a result, HOTAIR is essential for controlling the inflammatory response and macrophage glucose metabolism (184, 185). RA patients’ PBMCs and serum exosomes exhibit higher HOTAIR expression, whereas differentiated osteoclasts and rheumatoid synoviocytes exhibit lower HOTAIR expression. Through the regulation of miR-138, lentiviral overexpression of HOTAIR decreased the levels of IL-17, IL-23, IL-1β, and TNFα and prevented NF-κB activation in chondrocytes treated with LPS (180, 186, 187). On the other hand, HOTAIR expression in cardiomyocytes dramatically rose in a mouse model of sepsis. In addition to lowering TNFα synthesis and p65 phosphorylation in cardiomyocytes, silencing HOTAIR enhanced cardiac function in septic mice (180, 188).

Adenovirus-mediated overexpression of liver-expressed lncRNA (LeXis) in the liver, using a thyroxine-binding globulin promoter, reduced aortic lesion size and total serum cholesterol levels, confirming its role in maintaining hepatic sterol content (180). Another lncRNA is RP5-833A20.1, which reduces cholesterol efflux and increases inflammatory cytokines, including IL-1β, IL-6, and TNFα in THP-1 macrophages. Mechanistically, RP5-833A20.1 decreases the expression of NFIA by inducing miR-382–5p expression (189). Moreover, NEAT1-/- mice reveal NEAT1 as a novel lncRNA immunoregulator influencing monocyte-macrophage functions and T-cell differentiation. It is involved in a deregulated molecular circuit with various chemokines and interleukins post-MI, which could help identify high-risk patients for immunomodulatory therapies (190). Another example is ANRIL, whose silencing led to an elevation in the expression levels of the antisense transcripts p15ARF and p16INK4b, which are crucial regulators of senescence, apoptosis, and self-renewal of stem cells through the retinoblastoma-p53 pathway by disrupting PRC-1/2 attachment to their respective loci. Additionally, ANRIL directly binds to PRC-1/2 components like SUZ12 and CBX7, highlighting its function in epigenetic regulation. Contradictory results may result from isoform-specific effects caused by its multiple splice sites (180, 191).

Additionally, lncRNAs have the feature of acting as a sponge for miRNAs, masking their binding to their target mRNAs and therefore influencing gene expression and may regulate miRNAs as competitive endogenous RNAs (ceRNAs), contributing to autoimmune diseases (192–194). For instance, lncRNA HIX003209 enhances macrophage-mediated inflammation via TLR2/TLR4 and thereby contributes to the pathogenesis of RA. Through the IκBα/NF-κB signaling pathway, it stimulates the activation and proliferation of macrophages. By binding to miR-6089, lncRNA HIX003209 functions as a competing endogenous RNA (ceRNA), reviving TLR4 expression and stimulating NF-κB in macrophages (194). lincRNA-cox2 governs the release of pro-inflammatory cytokines TNF-α and IFN-γ, which have a significant impact on immune response (170).

### 4.3 Role of circRNAs in inflammation

circRNAs play a role in inflammation and cancer by modulating cellular components and influencing cancer-related inflammatory signaling pathways, often acting as miRNA sponges to activate or repress these pathways (195–197). They may function by binding to cRBPs and by encoding translatable peptides that modulate disease-related signaling pathways. They also interact with the PI3K/AKT pathway primarily through competing endogenous RNAs that sponge miRNAs, activating or repressing downstream pathways (196, 198). circHIPK3 was found to increase Foxo3a expression, functioning as a miR-421 sponge to inhibit inflammation and prevent ischemic injury (199). circHIPK3 expression was upregulated in synovial fluid mononuclear cells (SFMCs) from gouty arthritis patients. It enhances TLR4 expression and the NLRP3 inflammasome by sponging miRNAs, thus promoting inflammation in gouty arthritis (200). Pro-inflammatory cytokines are released into the TME and IME by NLRP3 inflammasomes, which NF-κB activates. This promotes the pathogenesis of inflammatory diseases and signaling pathways linked to cancer. Tumor and immune cells affect the initiation and spread of cancer, making the TME more difficult by facilitating metastasis, proliferation, and evasion. While TAMs, TANs, CAFs, MDSCs, and Tregs contribute to immunosuppression, T lymphocytes, B lymphocytes, natural killer, and dendritic cells are essential for tumor suppression (196).

Circular ANRIL (cANRIL), which is produced by exon skipping, disrupts pre-rRNA processing and ribosome biogenesis in SMCs and macrophages by binding to PES1, a crucial component of 60S preribosomal assembly. Like linear ANRIL, this causes nucleolar stress and p53 activation, which inhibits proliferation and promotes apoptosis (201, 202). Additionally, circ_0001005 and Exo-circGSE1 are prominent instances of interacting with the TME, encouraging immune system escape by altering immunity checkpoints such as PD-L1 (203). More examples of miRNAs, lncRNAs, circRNAs, and their role in inflammation are summarized in **Table 2**.

**Table 2:** Key non-coding RNAs (ncRNAs) involved in inflammation: inflammatory role and target pathways.

ncRNA Type	ncRNAs	Inflammatory Role	Pathway	References
miRNA	miR-146a	Anti-inflammatory	TNF, NF-κB and MEK-1/2, and JNK-1/2	(160, 350)
	miR-21		PTEN and SPRY1, TNFR1, TNFR2	(156, 351, 352)
	miR-223		NF-κB, Keap1, NLRP3, Nrf2	(353, 354)
	miR-29-3p		PI3k/Akt/FOXO3	(355)
	Let-7		LIN28/let-7	(356)
	miR-155	Pro-Inflammatory	TLR-mediated signaling pathway, PI3K-AKT, JNK	(152, 357–359)
	miR-124		DACT1 and activating the Wnt/β-catenin, JAK/STAT pathway	(360, 361)
	miR-125b	Anti/pro-Inflammatory	MAPK, NF-ĸB, JAK/STAT	(147, 362)
lncRNA	ANRIL	Pro-Inflammatory	NF-κB, AdipoR1/AMPK/SIRT1	(363, 364)
	MALAT1		JAK/STAT, PI3k/Akt/GSK-3 beta	(365, 366)
	HOTAIR		Wnt/β-catenin, TGF-β	(184, 367, 368)
	lincRNA-p21		P53-dependent, JAK2/STAT3	(369–371)
	lincRNA-COX2		JAK3/STAT3, NF-κB	(181, 372)
	NEAT1		miR-9-5p and TGF-beta, FZD3/GSK3beta/P-tau, ERK1/2, AKT, TLR4, TRAF6	(373–375)
	TUG		ERK, GLUT4	(376–378)
circRNA	ciRs-7	Pro-Inflammatory	Intra-lariat splicing, EGFR/STAT3	(379, 380)
	circHIPK3		TLR4	(200, 381)
	circFGFR2		JNK/MAPK	(382, 383)
	circPVT1		Type I interferon (IFN) signaling pathways	(384)
	circZNF609	Anti-Inflammatory	Hedgehog pathways, ETV1, FOXM1 or GNB2	(385–387)

## 5 Therapeutic potential of ncRNA

### 5.1 Current status of ncRNA therapies

In recent years, there have been tremendous advancements in ncRNA therapies, with several potential candidates progressing through clinical trials and even gaining regulatory approval. To date, 11 RNA-based therapeutics in the form of either antisense oligonucleotides (ASOs) or small interfering RNAs (siRNAs) have been approved by the FDA and/or the European Medicines Agency (EMA) (204). The RNA therapeutics targeted diseases include hereditary transthyretin amyloidosis, primary hyperoxaluria type 1, and Duchenne muscular dystrophy, and most of these therapies are delivered subcutaneously (targeting the liver). In contrast, the minority are delivered intravenously (targeting the liver or muscle) (**Table 3**). Despite currently having only ASOs and siRNAs on the market, multiple novel RNA therapeutics, such as miRNA mimics and anti-miRNAs, are in clinical development phase II or III clinical development (**Table 3**). At the same time, several other RNA-based therapies are being researched, such as therapeutic circRNAs, miRNA sponges, short hairpin RNAs (shRNAs) and CRISPR-Cas9-based gene editing (205–207). The use of miRNA therapies has multiple potential advantages over the currently approved ASO and siRNAs. Because miRNAs target a plethora of genes by nature, their effect is multifactorial while being specific in the case of targeting multiple genes in the same pathway. A good example of this is miR-21 which has been shown to target multiple genes in the PI3K-Akt pathway, such as PTEN and PIK3R1 (74, 208). This powerful effect of targeting multiple downstream genes presents a huge potential in boosting therapeutic effects; however, it does not come without drawbacks, as multiple targets also lead to off-target effects, which brings us to the problem of biological and technical challenges faced by ncRNA therapies research and development.

**Table 3:** List of non-coding RNAs (ncRNAs) therapies in the market and in clinical trials.

Name	Type	Modification and Delivery	Route of Administration	Target Organ	Disease	Status	Clinicaltrials.gov ID
Fomivirsen	ASO	Phosphorothioate	Intravitreal	Eye	Cytomegalovirus (CMV) retinitis in immunocompromised patients	FDA (1998) and EMA (1999) approved	N/A
Mipomersen	ASO	2'-O-methoxyethyl gapmer	Subcutaneous	Liver	Homozygous familial hypercholesterolemia	FDA (2013) and EMA (2012) approved	N/A
Nusinersen	ASO	2'-O-methoxyethyl	Intrathecal	Central nervous system	Spinal muscular atrophy	FDA (2016) and EMA (2017) approved	N/A
Eteplirsen	ASO	2'-O-methoxyethyl PMO	Intravenous	Muscle	Duchenne muscular dystrophy	FDA (2016) approved	N/A
Inotersen	ASO	2'-O-methoxyethyl; GalNAc	Subcutaneous	Liver	Hereditary transthyretin amyloidosis	FDA and EMA (2018) approved	N/A
Patisiran	siRNA	2′-fluoro/2′-O-methyl; liposomal	Intravenous	Liver	Hereditary transthyretin amyloidosis	FDA (2019) and EMA (2018) approved	N/A
Golodirsen	ASO	2'-O-methoxyethyl PMO	Intravenous	Muscle	Duchenne muscular dystrophy	FDA (2019) approved	N/A
Givosiran	siRNA	2′-fluoro/2′-O-methyl; GalNAc	Subcutaneous	Liver	Acute hepatic porphyria	FDA (2019) and EMA (2020) approved	N/A
Viltolarsen	ASO	2'-O-methoxyethyl PMO	Intravenous	Muscle	Duchenne muscular dystrophy	FDA (2020) approved	N/A
Volanesorsen	ASO	2’-O-methoxyethyl gapmer	Subcutaneous	Liver	Familial chylomicronaemia syndrome	EMA (2019) approved	N/A
Inclisiran	siRNA	2′-fluoro/2′-O-methyl; GalNAc	Subcutaneous	Liver	Atherosclerotic cardiovascular disease, elevated cholesterol, homozygous/heterozygous familial hypercholesterolaemia	EMA (2020) approved	N/A
Lumasiran	siRNA	2′-fluoro/2′-O-methyl; GalNAc	Subcutaneous	Liver	Primary hyperoxaluria type 1	FDA (2020) and EMA (2020) approved)	N/A
rAAV5-miHTT	Pri-miR-451 backbone	Adeno-associated viral vector (AAV5)	Intrastriatal	Brain	Huntington disease	Phase I/II	NCT04120493
WVE-120102	ASO	N/A	Intrathecal	Brain	Huntington disease	Phase I/II	NCT03225846, NCT04617860
RG-125	Anti-miR-103/107	GalNAc antagomiR	Subcutaneous	Liver	Type II diabetes, nonalcoholic fatty liver disease	Phase I/II	NCT02612662, NCT02826525
Remlarsen	miR-29 mimic	Cholesterol conjugated	Intradermal	Skin	Keloid (pathological fibrosis)	Phase II	NCT02603224, NCT03601052
siG12D-LODER	siRNA	Biodegradable polymeric matrix (PLGA)	Intratumoral	Tumour	Advanced pancreatic cancer	Phase II	NCT01188785; NCT01676259
Prexigebersen	ASO	Liposomal	Intravenous	Blood and/or immune cells	Acute myeloid leukaemia, chronic myeloid leukaemia	Phase II	NCT01159028; NCT04196257; NCT02781883
Olpasiran	siRNA	GalNAc	Subcutaneous	Liver	Cardiovascular disease	Phase II	NCT03626662, NCT04270760
Vupanorsen	ASO	GalNAc	Subcutaneous	Liver	Dyslipidaemias, hyperlipidaemias, hyperlipoproteinaemias	Phase II	NCT04459767, NCT03371355, NCT04516291
Miravirsen	Anti-miR-122	Phosphorothioate; LNA gapmer; Liposomal	Subcutaneous	Liver	Hepatitis C virus infection	Phase II	NCT01646489, NCT01727934, NCT01872936, NCT01200420
Donidalorsen	ASO	GalNAc; Phosphorothioate;2’-O-methoxyethyl	Subcutaneous	Liver	Hereditary angio-oedema, COVID-19	Phase II	NCT03263507, NCT04030598, NCT04307381, NCT04549922
BMT 101	siRNA	Carrier-free	Intradermal	Skin	Hypertrophic scar	Phase II	NCT03133130, NCT04012099
Danvatirsen	ASO	GalNAc	Intravenous	Tumour	Metastatic NSCLC, resectable early-stage NSCLC, pancreatic cancer, mismatch repair-deficient colorectal cancer	Phase II	NCT03819465, NCT03794544, NCT02983578
Bamosiran	siRNA	Carrier-free	Topical	Eye	Ocular hypertension, glaucoma	Phase II	NCT00990743, NCT01227291, NCT01739244, NCT02250612
Cemdisiran	siRNA	GalNAc	Subcutaneous	Blood	Paroxysmal nocturnal haemoglobinuria, IgA nephropathy, Berger disease, glomerulonephritis	Phase II	NCT04601844, NCT02352493, NCT03841448, NCT03999840
Apatorsen	ASO	2'-O-methoxyethyl gapmer	Intravenous	Tumour	Squamous cell lung cancer, non-squamous NSCLC, urological neoplasms, metastatic bladder cancer, urinary tract neoplasms, castration-resistant prostate cancer	Phase II	NCT01120470, NCT01454089, NCT01829113, NCT02423590
Sepofarsen	ASO	Chemically modified	Intravitreal	Eye	Leber congenital amaurosis type 10 (LCA10), blindness, LCA, vision disorders, sensation disorders, neurological manifestations, eye diseases, hereditary or congenital eye diseases	Phase II/III	NCT03140969, NCT03913143, NCT03913130
AKCEA-TTR-LRx	ASO	GalNAc	Subcutaneous	Liver	Hereditary transthyretin-mediated amyloid polyneuropathy	Phase III	NCT04302064; NCT03728634; NCT04136184; NCT04136171
Alicaforsen	ASO	Phosphorothioate	Oral	Intestine	Crohn’s disease	Phase III	NCT03473626, NCT00063830, NCT00063414, NCT00048113, NCT02525523
Nedosiran	siRNA	GalNAc	Subcutaneous	Liver	Primary hyperoxaluria type 1 and primary hyperoxaluria type 2, kidney diseases, urological diseases	Phase III	NCT03392896, NCT04555486, NCT04580420, NCT03847909, NCT04042402
Pelacarsen	siRNA	GalNAc	Subcutaneous	Liver	Hyperlipoproteinaemia	Phase III	NCT03070782, NCT03070782, NCT04023552
Tivanisiran	siRNA	Phosphorothioate; 2'-O-methoxyethyl	Topical	Eye	Dry eye disease	Phase III	NCT01438281, NCT01776658, NCT02455999, NCT03108664
Tominersen	ASO	Phosphorothioate; 2'-O-methoxyethyl gapmer	Intrathecal	Brain	Huntington disease	Phase III	NCT02519036, NCT04000594, NCT03342053, NCT03761849, NCT03842969

ASO, anti-sense oligonucleotide; LNA, locked nucleic acid; PMO, phosphoramidate morpholino oligomers; N/A, not available.

### 5.2 ncRNA therapeutics targeting cancer

#### 5.2.1 Therapeutics with completed trials

##### 5.2.1.1 APN401

APN401 is a cell therapy that silences Casitas-B-lineage lymphoma protein-b (Cbl-b) in PBMCs by isolating and transfecting them with a siRNA against Cbl-b before infusing them back into the patients. Cbl-b is a protein that acts as a checkpoint in several immune cells, and inhibiting it was shown to enhance immune cell antitumor activity *in vitro* human cells and animal trials. There were several clinical trials on this drug (up to phase Ib). So far, it has been demonstrated to be safe, increase the antitumor activity of the immune cells, and stabilize the condition of some patients with advanced solid tumors (209, 210).

##### 5.2.1.2 STP705

STP705 is a siRNA drug. It contains two types of siRNA molecules complexed with a histidine–lysine co-polymers platform (22). The histidine part allows the complex to lysis endosomes, and the lysine part allows the complex to bind the RNA. The two siRNA in the drug silence TGF-β1 and COX-2 genes, which are involved in several cancer-related pathways, including mediating drug resistance (211–213). There are several clinical trials on STP705, including two phase II trials completed for the use of the drug against squamous cell carcinoma (*in situ*) skin cancer (isSCC) [Clinicaltrials.gov ID: NCT04844983] and cutaneous SCC [Clinicaltrials.gov ID: NCT04293679] with favorable safety and therapeutic outcomes (214, 215).

##### 5.2.1.3 TBI-1301

TBI-1301 is a cell therapy technique in clinical trials against several cancers expressing NY-ESO-1 (a cancer marker). Some T-cells are taken from the patient and genetically modified to express a T-cell receptor (TCR) specific to NY-ESO-1 and a siRNA to silence endogenous TCR before being administered back to the patients. The phase I/II clinical trial [Clinicaltrials.gov ID: NCT03250325] done on this drug was done on eight patients with synovial sarcoma who are surgically unresectable and refractory to anthracycline therapy. Four patients showed partial response to the drug. There is currently another active phase I clinical trial for the same treatment against solid tumors that is estimated to be completed in 2025 [Clinicaltrials.gov ID: NCT02869217].

##### 5.2.1.4 NU-0129

NU-0129 is a therapeutic in clinical trials (Early Phase 1) [Clinicaltrials.gov ID: NCT03020017] intended to treat glioblastoma. It is an interfering RNA (RNAi) based spherical nucleic acid (SNA). This structure consists of a spherical gold nanoparticle in the middle, coated with oligoethylene glycol (OEG) or polyethylene glycol (PEG). It has siRNA attached to it in a dense and ordered pattern. This structure can pass the blood-brain barrier (BBB) and the blood-tumor barrier (BTB) and accumulate in the tumor. The siRNA targets the Bcl2Like12 (Bcl2L12) gene. The gene, when active, prevents cancer cells from apoptosis. The study was done on eight patients with glioblastoma multiforme (GBM) or gliosarcoma (GS) who were going to have surgery to remove the tumor (subtotal or gross total resection), which will then be further studied. The results of the study showed that only one patient had a serious adverse event (that was not related to the drug), that gold nanoparticles were found in the tumor tissue and inside the tumor cells, and that there is a correlation between the accumulation of the particles and the inhibition of Bcl2L12 protein (21).

##### 5.2.1.5 LNA-i-miR-221

LNA-i-miR-221 is a nucleic acid molecule that targets miR-221 involved in controlling many aspects related to malignancy in several cancers (216). The molecule of the drug has its ribose sugar modified to be a locked nucleic acid (LNA) where the 2’ oxygen and 4’ carbon are covalently linked together. This increases the stability of the RNA to degradation and increases the affinity to its target. The backbone is also modified using phosphorothioate chemistry, where an oxygen atom on the phosphate group in the normal backbone is substituted with a sulfur atom. Which also increases stability (20). LNA-i-miR-221 is a 13-mer that binds to miR-221 and inhibits it. The clinical study (phase I) [Clinicaltrials.gov ID: NCT04811898] included 17 multiple myeloma patients (only 16 continued to further analysis). The results showed that the drug had a good safety profile. Eight patients showed stable disease, and one patient showed a partial response. In addition, pharmacodynamic studies showed decreased expression of miR–221 in PBMC (217).

##### 5.2.1.6 NBF 006

NBF 006 is a siRNA drug that is encapsulated in a lipid nanoparticle. It targets Glutathione S-transferase Pi (GSTP), which is a gene that is involved in oncogenes regulation and chemoresistance (218). The phase I clinical trial [Clinicaltrials.gov ID: NCT03819387] was done on patients with NSCLC, CRC, or pancreatic cancer. The study was divided into a dose escalation part and a dose expansion part. The trial showed no level 4 or 5 adverse effects, and no serious adverse events or dose-limiting toxicities were observed. PBMCs tested after the treatment showed reduced GSTP mRNA, and out of 38 NSCLC patients, 2 had a partial response, and 17 had a stable disease outcome (219).

#### 5.2.2 Therapeutics that are in active clinical trials or recruiting

##### 5.2.2.1 EphA2 siRNA

EphA2 siRNA is a siRNA drug that targets the EphA2 gene. There is currently a phase 1 clinical trial [Clinicaltrials.gov ID: NCT01591356] running that is expected to be completed by 2025. The study enrolled 49 patients with advanced tumors. The primary objective of this study is to determine the toxicity profile and the maximal tolerated dose of the drug. EphA2 is a receptor tyrosine kinase that is overexpressed in many types of cancer and is involved in the regulation of tumorigenesis. There are several clinical trials for drugs that target this gene (220). The siRNA is incorporated into 1,2-Dioleoyl-sn-Glycero-3-Phosphatidylcholine (DOPC) liposomal particles, which is a neutrally charged liposome particle (23).

##### 5.2.2.2 KrasG12D

This drug is made of exosomes with KrasG12D siRNA derived from stromal cells. Phase 1 clinical trial is currently running, with 15 enrolled patients with pancreatic ductal adenocarcinoma harboring KrasG12D mutation. The study aims to determine the maximum tolerated dose, minimal residual disease rate in high-risk patients, overall survival, and progression-free survival [Clinicaltrials.gov ID: NCT03608631]. KRAS is the most frequently mutated oncogene and is involved in the regulation of many signaling pathways involving many cell functions, including cell survival and cell cycle progression. It naturally flips from an active to an inactive state depending on a variety of variables. Common mutations to this gene that are found in cancers are in codons 12 and 13, with G12D mutation being the most common in pancreatic adenocarcinoma. The mutation makes the protein active all the time (221). Using exosomes as a delivery vehicle for siRNA shows efficient cell uptake and decreased clearance compared to liposomes (222).

##### 5.2.2.3 SLN124

SLN124 is a drug that is in a clinical trial (phase 1/2) [Clinicaltrials.gov ID: NCT05499013] for the treatment of patients with polycythemia vera (PV), which is a kind of blood cancer that results in increased levels of RBCs. Patients need recurrent therapeutic phlebotomies to decrease the risk of thrombosis. Most patients with PV have iron deficiency, and the treatment with therapeutic phlebotomies further increases the problem, which increases the need for therapeutics that target the underlying iron distribution problem (223). SLN124 is a siRNA targeting transmembrane protease, serine 6 (TMPRSS6)), which negatively regulates hepcidin, an important protein in iron homeostasis. Upregulating hepcidin by inhibiting TMPRSS6 using antibodies or by using hepcidin mimetics was shown to improve the condition in animal models (223). The clinical trial aims to enroll 65 patients and determine the drug’s safety and efficacy. In phase II part of the study, a placebo is used in one group of patients as a control group, while the other group is treated with SLN124.

##### 5.2.2.4 MTL-CEBPA

MTL-CEBPA is a small activating RNAs (saRNAs) drug that targets the upregulation of CCATT/enhancer binding protein alpha (CEBPA). saRNAs can activate genes by binding to Ago proteins to form a complex that targets promoters (224). CEBPA is an important transcription factor that is involved in hepatocyte homeostasis, and several studies related its downregulation to liver fibrosis or cancer (225, 226). MTL-CEBPA is several clinical trials in combination with other anticancer drugs such as PD-1 [Clinicaltrials.gov ID: NCT04105335], sorafenib [Clinicaltrials.gov ID: NCT02716012, NCT04710641], atezolizumab and bevacizumab [Clinicaltrials.gov ID: NCT05097911]. All the studies are in phase I except [Clinicaltrials.gov ID: NCT04710641], which is in phase II.

### 5.3 ncRNA therapeutics targeting inflammatory diseases

#### 5.3.1 Therapeutics with completed trials

##### 5.3.1.1 Tivanisiran

Tivanisiran is a siRNA drug that is formulated as an eye drop. It completed its phase 3 clinical trial in 2023 [Clinicaltrials.gov ID: NCT04819269] for its use in treating patients with dry eye disease with sjögren’s syndrome. No results were posted on the trail. 203 patients were enrolled in the study and were divided into two groups. One was a control group where they were treated only by the vehicle as a placebo, and the other group was treated using Tivanisiran. The treatment was once daily for three months. Another phase III clinical study [Clinicaltrials.gov ID: NCT05310422] was also completed. 301 patients with dry eye disease were involved in the trial. They were treated daily with Tivanisiran for a year. They were randomly divided into a control group treated with the vehicle as a placebo and a treatment group. In a press release about the trial, Sylentis, the company developing the drug, reported no significant differences in adverse effects between both groups, reflecting a favorable safety profile (227). Dry eye disease is an inflammatory disease that results in abnormalities in the precorneal tear film (228). The siRNA in Tivanisiran aims to silence the Transient Receptor Potential Vanilloid 1 (TRPV1) gene. TRPV1 is involved in pain transduction and innate inflammatory response (229).

#### 5.3.2 Therapeutics that are in active clinical trials or recruiting

##### 5.3.2.1 siSPARC/siLR4A microneedle patch

In this phase II study [Clinicaltrials.gov ID: NCT06138964], siRNA microneedle patch is used to treat scar tissues. The effect of using siRNA against SPARC is compared to using a mixture of 2 siRNA molecules against SPARC and ILR4A. The study aims to recruit 50 patients with post-surgical Scars. The primary aim is to study both groups’ efficacy in reducing post-surgical scar elevation. SPARC is an extracellular matrix protein that is overexpressed in keloid scar tissues. Overexpressing it in human keloid fibroblasts *in vitro* increases cell proliferation, migration collagen production, and extracellular matrix synthesis (230). ILR4A is a protein expressed in several immune cells. It plays a role in wound healing and fibroblast proliferation. It was shown to be overexpressed in people with familial histories of keloid scars (231). Another research highlighted that the IL-4/IL-13 axis plays a role in scar tissue formation (232).

##### 5.3.2.2 AZD6912

AZD6912 is a siRNA therapy in a phase I clinical trial [Clinicaltrials.gov ID: NCT06115967] developed to treat inflammatory arthritis. AZD6912 targets the Complement Factor B (CFB) gene, which is part of the complement activation pathway that plays a role in the initiation and progression of RA (233, 234). The study aims to recruit 64 healthy individuals, with the primary aim of determining the drug’s safety and tolerability.

## 6 Clinical applications of ncRNA biomarkers

### 6.1 Early detection and diagnosis

Because of the presence and stability of ncRNA biomarkers in body fluids such as blood, urine, or saliva, they began to revolutionize clinical applications for early disease detection, including cancer and inflammatory diseases. Previous research reported that the expression of peripheral lncRNA MALAT1 was measured in patients with NSCLC and healthy subjects. MALAT1 was upregulated in NSCLC patients, providing a foundation for the possibility of MALAT1 as a biomarker assisting in distinguishing this disease at early stages. Moreover, the combination of MALAT1 and other established markers, such as carcinoembryonic antigen (CEA), could play a valuable role in enhancing the diagnostic performance of either marker. Additionally, lncRNAs such as SPRY4-IT1, ANRIL, and NEAT have high accuracy in detecting NSCLC (235, 236).

Different expression levels of ncRNAs, especially miRNAs, in inflammatory diseases indicate the progression of such diseases, including inflammatory bowel disease and RA (147). Researchers investigated the role of HOTAIR in inflammation regulation within macrophages. When NF-κB was suppressed, several long non-coding inflammation-associated RNAs (linfRNAs), including HOTAIR, decreased. HOTAIR was shown to alleviate RA symptoms by targeting miR-138, inhibiting inflammation pathways (237). This suggests that ncRNAs like HOTAIR could be used as non-invasive tests for early detection of inflammatory diseases (184). The knockdown of linfRNA1 also reduced cytokine levels, further indicating its potential as a biomarker for diagnosing inflammatory conditions (237). In another context, researchers explored ncRNAs for their predictive value and role in the early detection of preeclampsia during pregnancy. A study identified certain circulating small ncRNAs expressed differentially between women who developed preeclampsia and those with normal pregnancies, highlighting their potential for early diagnosis (238).

Emerging diagnostic technologies like liquid biopsy highlight ncRNAs’ role in early cancer detection. Liquid biopsy analyzes circulating tumor DNA and ncRNAs from a simple blood draw, minimizing the need for invasive procedures. Circulating ncRNAs can distinguish between cancerous and non-cancerous cases with high sensitivity (238). Single-cell RNA sequencing (scRNA-seq) also detects gene expression at the single-cell level, identifying unique ncRNA signatures associated with specific cancer subtypes or stages, aiding early cancer diagnosis (239). Studies also demonstrate the potential of circulating lncRNAs as non-invasive biomarkers for CRC. Specific lncRNAs, such as XLOC006844 and LOC152578, showed stable expression in blood plasma and achieved sensitivity and specificity rates of 80% and 84%, respectively, in CRC detection. These findings suggest that circulating ncRNAs can improve compliance rates for CRC screening compared to traditional methods like colonoscopy (240).

A comprehensive analysis involving multiple studies highlighted the diagnostic efficacy of circulating miRNAs for HCC. A panel comprising miR-122, miR-192, and miR-21 achieved good results distinguishing HCC from chronic hepatitis B and cirrhosis, demonstrating how integrating ncRNA biomarkers enhances diagnostic sensitivity beyond traditional methods (238, 241). Research on OSCC identified a six-lncRNA diagnostic risk model with remarkable specificity and sensitivity in differentiating OSCC samples from normal tissues, showcasing the potential of lncRNAs in predictive models for early cancer detection (239). Altered expressions of miR-181 and miR-652 were also associated with advanced GC stages, highlighting their diagnostic potential in distinguishing early and late-stage disease (242).

### 6.2 Prognostic and predictive biomarkers

Recently, scientists have explored the potential of ncRNAs as biomarkers for cancer prognosis and therapy response. ncRNAs play key roles in tumor behavior and patient outcomes. A study identified a lncRNA signature predicting outcomes and recurrence in stage II CRC, outperforming conventional clinicopathological data like CEA levels or T Stage. This panel allowed personalized treatment and improved survival outcomes (243). A meta-analysis demonstrated the predictive power of lncRNA signatures integrated with clinical risk factors, such as breast cancer. For instance, low levels of miR-206 were associated with poor overall survival and tumor progression, while overexpression of miR-1225 was linked to unfavorable prognostic markers, indicating their use in disease progression monitoring (244).

Novel miRNAs, such as MSM2 and MSM3, show promise as therapeutic targets due to their roles in cancer cell proliferation and migration. These miRNAs may also serve as prognostic indicators for cancer progression (245). Changes in lncRNA expression profiles can predict disease activity flares, enabling timely treatment adjustments (237). Dysregulated lncRNAs in SLE correlate with disease severity and clinical parameters like the erythrocyte sedimentation rate and autoantibody levels, aiding in disease monitoring (246). ncRNAs also contribute to the pathogenesis of multiple sclerosis, where specific miRNA profiles indicate disease activity and relapse rates, allowing real-time monitoring (247). Similarly, miRNA expression changes in Sjögren’s syndrome are linked to disease activity and response to therapies like interferon-beta, reinforcing their role in monitoring autoimmune diseases (248).

A lncRNA-based signature stratified CRC patients into high- and low-risk groups for recurrence-free survival, validating the model’s predictive accuracy (243). Another study found miR-93 effective as a prognostic biomarker for breast cancer, offering high sensitivity and specificity for non-invasive disease monitoring (244). In lung cancer, specific miRNAs like miR-21 are linked to poor prognosis in NSCLC, demonstrating their dual role in diagnosis and treatment prediction (249). A meta-analysis confirmed the prognostic value of ncRNAs for breast cancer, noting that high miRNA expression levels often correlate with poor survival and increased metastasis risks, particularly with elevated PVT1 levels (250). The lncRNA MALAT1, strongly associated with triple-negative tumor types in lung and breast cancer, also emerged as a significant predictor for disease-free and overall survival (251).

### 6.3 ncRNAs and personalized medicine

ncRNAs play a pivotal role in personalized medicine due to their biomarker and therapeutic target roles. Integrating ncRNA profiling into personalized medicine represents a transformative healthcare approach. Clinicians can tailor therapeutic strategies using ncRNA expression patterns, which are more effective than traditional modalities (252). For instance, circulating miR-139-5p correlates with improved survival rates in radiotherapy patients, enabling personalized treatment monitoring (253). Therapeutic approaches targeting ncRNAs, such as miRNA mimics or inhibitors, restore normal gene expression patterns disrupted in diseases. In lung cancer, anti-miR-155 therapy resensitized cancer cells to chemotherapy, highlighting how an individual’s ncRNA profile can guide personalized treatment strategies (207). Clinical trials incorporating ncRNA profiling have also shown promise. For example, a trial investigating miR-34a mimics with standard chemotherapy in lung cancer demonstrated that patients with specific miRNA expression profiles responded better, underscoring ncRNA’s potential in personalized approaches (254).

Chemoresistance remains a significant challenge, but developing miRNAs to counter resistance shows promise. For instance, miR-451a regulates chemotherapy resistance in gallbladder tumors (255). Also, HOTAIR predicts responses to neoadjuvant chemotherapy in breast cancer, where high expression levels are associated with poorer prognosis and treatment resistance. This highlights HOTAIR’s potential as a predictive biomarker for chemotherapy efficacy (250). Another example is lncARSR. Elevated lncARSR levels, associated with sunitinib resistance in renal cell carcinoma, were linked to poor outcomes. Targeting lncARSR reversed resistance mechanisms and improved treatment efficacy, emphasizing ncRNA profiling’s clinical relevance (256). Innovative treatment modalities targeting ncRNAs, such as ASOs, are under development. ASOs selectively bind to target lncRNAs, preventing their pathological effects. Promising results have been seen with ASOs targeting lncRNAs in breast cancer progression, offering a new avenue for personalized treatments (207, 252).

## 7 Challenges and future directions

### 7.1 Biological and technical challenges

#### 7.1.1 Specificity

While there is potential in developing new therapies, many challenges hinder the process, with the main ones being specificity, delivery, and tolerability. ncRNAs usually regulate many target genes. Any off-target effect can lead to uncalculated consequences, potentially exacerbate diseases, or cause toxicities. The target genes also differ in their abundance and availability depending on the cell type in question. It has been reported that miRNAs could have contradicting roles in different cell types, such as miR-21 example, which has been widely studied for its oncogenic role in various cancers and has been considered a potential therapeutic target in cancer therapy, but it also has pro-immune roles such as activating roles in CD4+ and CD8+ T cells (257, 258). This specificity issue highlights the importance of rigorous preclinical evaluation and the need for refined targeting strategies to avoid not only the drug reaching off-target cell types but also reaching the correct targets within the target cell. Since RNA is negatively charged, it is unstable, and crossing cell membranes is difficult; therefore, chemical modifications are used to ameliorate the pharmacodynamics and pharmacokinetics (259, 260). First-generation modifications aim to enhance nuclease resistance and cellular uptake by replacing the phosphodiester bonds in the backbone of the molecule with phosphorothioate bonds (261). The first RNA therapy approved for clinical application in 1998 was using the first-generation modification in the drug Formivirsen, an ASO used in the treatment of rhinitis caused by the cytomegalovirus by targeting the cytomegalovirus IE2 mRNA (**Table 3**). As for the second-generation modifications, they enhance the nuclease resistance and target affinity of RNA molecules while also decreasing the accompanied toxicity and immune response and being effective in ASO, siRNAs, and miRNA mimics or inhibitors (262). Approved RNA therapeutics that use second-degree modifications include Lumasiran and Inclisiran (**Table 3**). Usually, in the sugar backbone, the 2’-O alkyl group is replaced with 2’O fluoro, 2’-O methyl, or 2’-O-methoxyethyl. Additionally, chimeric molecules with central regions of unmodified nucleotides flanked with the 2’-O modifications are called gapmers. They are usually synthesized to have a good target affinity along with an increased nuclease resistance (263). The third-generation modifications are built around a modification of the furanose ring that results in the creation of what are called locked nucleic acids or LNAs, phosphoramidate morpholino oligomers (PMOs) or peptide nucleic acids (PNAs) (264–268). These modifications enable the ncRNAs to have a significantly higher target affinity compared to the first-generation modification. The currently licensed RNA therapies, except the previously mentioned Formivirsen, have undergone second or third-generation chemical modifications (**Table 3**).

#### 7.1.2 Immunogenicity

The issue with ncRNA therapeutics and immunogenicity is that our immune system recognizes single-stranded as well as double-stranded RNA as exogenous due to mechanisms built for viral infection defense. This immune response is mediated by TLRs, which results in nuclear factor-κB (NF-κB) activation and the upregulation in the expression of cytokines that initiate an inflammatory response (IL-6, IL-8, IL-12 and TNF) (269). The previously mentioned second and third-generation chemical modifications were developed not only to improve specificity but also to reduce the immunogenicity of ncRNA therapies. Even with major improvements, clinical trials using these modified reagents continue to report negative immune responses. For example, MRX34, a miR-34a mimic, had demonstrated encouraging preclinical outcomes in terms of efficacy and safety however the clinical trial was stopped because five patients experienced side effects that are immunological, including cytokine release syndrome, systemic inflammatory response syndrome, and hepatic failure among other symptoms (270, 271). However, PMOs have been shown to reduce the immune response by exhibiting a neutralizing of the charge on ncRNA therapies, thus limiting their potential for protein interaction, such as with TLRs. Eteplirsen, an ASO FDA-approved and used for the treatment of Duchenne muscular dystrophy, is synthesized with this modification, and up to date, there have been no reports of immunogenicity triggered by this drug (272).

#### 7.1.3 Delivery

Another significant hurdle in ncRNA therapy development is the effective delivery of ncRNA therapeutics. There are multiple layers to the delivery challenge since ncRNA therapy should be delivered to the correct organ and to the specific cell type of interest. Also, it should be able to penetrate the membrane of this cell efficiently. Given the inherent fragility of RNAs, not only are they prone to physiological degradation, but also their ability to cross the cellular membranes is quite limited. To overcome this problem, the development of more sophisticated delivery mechanisms is currently being investigated. For example, the most studied are lipid nanoparticles that are being researched for their ability to facilitate the entry into target cells via encapsulation of the RNA molecules of interest, and they have efficiently been utilized in the case of mRNA vaccines (273, 274). For the targeted delivery of ncRNAs, a variety of lipid-based vesicles, including microemulsions, liposomes, and lipid nanoparticles, are being explored (275). Liposomes are either neutral or cationic vesicular structures that get associated with the negatively charged RNA to create a lipoplex that not only protects against RNA degradation but also enhances cellular uptake. Secondary modifications by adding excipients to the lipoplex surfaces, such as polyethylene glycol (PEG), cholesterol, or hyaluronic acid can lessen the non-specific uptake by the mononuclear phagocyte system (the reticuloendothelial system) (276–279). Important considerations should be considered when selecting suitable nanoparticles for clinical application, such as size and surface characteristics. To further increase the precision of targeting nanoparticles loaded with miRNAs or siRNAs, the conjugation with cancer-cell-specific ligands has been explored in pre-clinical studies with promising results (280). Biodegradability after the release of cargo is also a required point of study to avoid potential toxicities (252, 281). Studies have explored the effect of the size of nanoparticles administered intravenously on biodegradability and have detected that bigger nanoparticles accumulate in organs such as the liver and lungs. In contrast, smaller ones are processed by the kidneys (282).

Utilizing viral vectors such as adenovirus, retrovirus, lentivirus, and adeno-associated viruses to deliver ncRNA efficiently is another solution that is being explored. The use of viral vectors is beneficial as they can transfer genes into different tissues and cause long-term expression. Additionally, natural tropism is the inherent ability of a virus to infect a specific cell type. For example, adenovirus naturally targets cells in the nervous system, while retrovirus typically infect dividing cells, and lentivirus target a wider range of cells, including immune cells or stem cells (283, 284). Viral tropism is an advantage in the case of wanting to increase the precision of targeted delivery of ncRNA therapeutics. Even though adenovirus and lentivirus viral vectors have shown success in preclinical and clinical studies in treating human disease, multiple studies have demonstrated that each of the viruses described can induce miRNA pathway dysregulation, such as downregulation of certain miRNAs, requiring evaluation of the side effects and safety (285, 286). Moreover, the innate immune response is another challenge that is brought up regularly in the clinical development of viral vectors in gene therapy and should be considered and resolved (287). Nonetheless, while there is much potential in liposomal nanoparticles or viral vector drug delivery, these approaches introduce critical risks such as immune responses or potential insertional mutagenesis while they are explored for potentially increasing therapeutic efficacy, their safety profile is still under investigation.

### 7.2 Future directions

Several promising directions could play pivotal roles in ncRNA therapy efficacy and advancement. The rapid expansion of the bioinformatics field, along with its genomic technologies, immensely helps in ncRNA research and enables scientists to understand the roles of ncRNAs. Of these advances, high-throughput sequencing is at the top of the list in identifying promising ncRNA candidates that could either serve as therapeutic targets or biomarkers. The advancement and accessibility of such screening methods also benefit the field of precision or personalized medicine. The recent advances in gene sequencing and gene editing enable the design of effective patient-specific cell therapies, and this trend has pushed for the development of treatment that would overcome the standard approaches. This trend pushed for the development of a treatment that would overcome the standard approaches and, as a result, improve therapy outcomes in clinical settings (288, 289). In cancer medicine, the stratification of patients through the biomarkers they exhibit, as well as diagnostics that accompany the patient profile, has become the standard for anti-cancer drug development. Soon, the study of nanoparticles will employ the same standard of stratification of the patient population to develop effective personalized medicine (290). The heterogeneity of many diseases, notably in cancer, is challenging in the development of a one-size-fits-all ncRNA therapy since the expression of ncRNAs, as well as their function, varies significantly due to genetic variabilities among patients. This heterogeneity means that a single ncRNA therapy for a certain disease may not be applicable or efficient across the broad population, and genomic profiling and bioinformatic tools will provide a much more accurate identification of the potential of a specific ncRNA being effective on a certain patient or not. Not only this, but also a screening of patient tumors for ncRNA is helping enable the identification of ncRNA signatures associated with certain diseases, and this helps in the establishment of novel biomarkers (291).

CRISPR-Cas9 offers a whole array of exciting possibilities in ncRNA research. It could allow for the precise targeting of ncRNA regulatory elements within ncRNAs, such as promoters of lncRNAs, or by modulating the expression levels of non-coding RNAs. However, in a study targeting lncRNAs in glioma cells, CRISPR interference (CRISPRi) was used to knock down thousands of lncRNAs and test the effect on radiation therapy sensitivity of glioma cells. The results were impressive, and a single lncRNA was identified that was found to significantly sensitize glioma cells to the treatment. Thus, a single lncRNA was identified and found to significantly sensitize glioma cells to the treatment. Thus this study demonstrated the potential of CRISPRi as a promising tool for identifying novel therapeutic targets (292). For the CRISPR–Cas9 system to be applied physiologically however, delivery challenges, especially regarding the immune response to the Cas9 proteins, must be carefully studied and overcome (207, 293). Finally, there is much promise in investigating combination therapies that incorporate ncRNA interventions with currently used treatments like immunotherapy or chemotherapy. In cancer and other complicated diseases, these combination approaches may improve therapeutic efficacy and reduce resistance mechanisms that frequently hinder treatment outcomes. Patient outcomes may be enhanced by sensitizing tumors to traditional treatments through the use of ncRNAs (294, 295). Much effort is also being exerted towards the creation of nanoparticles that can co-deliver various therapeutic compounds to target multiple genes in numerous cells: polymeric micelles and circulating nanoparticles with a pH-regulated drug release mechanism have demonstrated good initial results in preclinical studies (296, 297). Another promising approach is the development of bio-engineered lncRNA molecules capable of carrying multiple small RNAs at once, including siRNAs, antimiRs, and miRNA mimics. These constructs have shown a successful inhibition in the growth of multiple cancer cell lines *in vitro* and will probably be further explored in other contexts (298).

## 8 Conclusion

In summary, research has revealed the crucial roles of ncRNA in terms of gene regulation in contexts such as inflammation and cancer. It has become evident that miRNAs, lncRNAs, and circRNAs are integral players in the regulatory gene expression network as influencers of transcriptional, post-transcriptional regulation, and epigenetic regulation. In cancer, ncRNAs have been identified as oncogenes or tumor suppressors with expression levels that are often dysregulated in various cancers. Understanding their functions opens an avenue of opportunities to develop potential therapeutics, and their patterns of expression can be used as biomarkers for diagnosis and prognosis or prediction of disease recurrence. Similarly, in inflammatory diseases, ncRNAs play pivotal roles and can function as immune modulators through cytokine expression regulation, which highlights their potential as biomarkers and therapeutics to manage these conditions. The development of diagnostic techniques utilizing ncRNA profiles could revolutionize patient care through a non-invasive method of monitoring disease progression. However, despite the advancements reached in understanding ncRNAs, many mechanisms remain unclear and necessitate further research to fully elucidate their roles. Many challenges also face ncRNA therapeutics development, such as immunogenicity, specificity, and delivery, and they are still being optimized. As research progresses and our comprehension deepens, their integration into clinical practice holds promise to enhance diagnostic accuracy and therapeutic efficacy.

## Glossary

Epigenetic Alterations: Changes in gene function that do not involve changes in the DNA sequence, often through DNA methylation or histone modification
Splicing: A process by which introns are removed from a transcribed pre-mRNA and exons are joined together to form a mature mRNA sequence
Chromatin Remodeling: The dynamic modification of chromatin architecture to allow access of transcriptional machinery to DNA for gene expression regulation
CRISPR-Cas9: A gene-editing technology used for targeted DNA modification. In non-coding RNA research, CRISPR can modulate ncRNA expression levels or target ncRNA regulatory elements
Gene Silencing: The process of suppressing gene expression, often through RNA interference, which can be therapeutic by inhibiting genes involved in disease pathways
Tumor Microenvironment: The environment surrounding a tumor, including immune cells, blood vessels, and signaling molecules that can influence tumor progression or suppression
